# From domestication to imperial patronage: Deconstructing the biomedicalisation of occupational therapy

**DOI:** 10.1177/13634593211067891

**Published:** 2021-12-23

**Authors:** Pier-Luc Turcotte, Dave Holmes

**Affiliations:** Université de Sherbrooke, Canada; University of Ottawa, Canada

**Keywords:** deconstruction, Deleuze & Guattari, history, poststructuralism, resistance

## Abstract

Occupational therapy knowledge emerged in the 19th century as reformist movements responded to the industrialisation of society and capitalist expansion. In the Global North, it was institutionalised by State apparatuses during the First and Second World Wars. Although biomedicine contributed to the rapid expansion and establishment of occupational therapy as a health discipline, its domestication by the biomedical model led to an overly regulated profession that betrays its reformist ideals. Drawing on the work of Deleuze and Guattari, our aim in this article is to deconstruct the biomedicalisation of occupational therapy and demonstrate how resistance to this process is critical for the future of this discipline. The use of arts and crafts in occupational therapy may be conceptualised as a ‘nomad science’ aesthetically resisting the domination of industrialism and medical reductionism. Through the war efforts, a coalition of progressive nurses, social workers, teachers, artisans and activists metamorphosed into occupational therapists. As it did with nursing, biomedicine proceeded to domesticate occupational therapy through a form of ‘imperial’ patronage subsequently embodied in the evidence-based movement. ‘Occupational’ jargon is widely used today and may be viewed as the product of a profession trying to establish itself as an autonomous discipline that imposes its own regime of truth. Given the symbolic violence underlying this patronage, the future of occupational therapy should not mean behaving according to biomedicine’s terms. As a discipline, occupational therapy must resist the appropriation of its ‘war machine’ and craft its own terms through the release of new creative energy.

*The task of schizoanalysis is that of learning what a* subject’s *desiring-machines are, how they work, with what syntheses, what bursts of energy, what constituent misfires, with what flows, what chains, and what becomings in each case. Moreover, this positive task cannot be separated from indispensable destructions, the destruction of the molar aggregates, the structures and representations that prevent the machine from functioning.*

– Deleuze and Guattari (1972, p. 338)

## Introduction

The knowledge base of occupational therapy in the Global North was widely influenced by the use of arts and crafts in various resistance movements that developed in the 19th century in response to post-industrial, capitalist expansion and biomedical reductionism (Taff and Balubal, 2021). Prior to their use in Western medical contexts, creative arts and crafts were frequently cited as healing and caring methods in most pre-colonial societies (Jhaveri, 2020) and contributed to their own cultural survival and resistance to colonisation (Archibald and Dewar, 2010). By the end of the 1800s and early 1900s, the use of occupation as a ‘treatment for the insane’ in psychiatric asylums was conceived of as involving crafts and manual work (Moher, 1907). In this context, work did not necessarily mean paid employment (Harvey-Krefting, 1985) and was mostly proposed as a treatment prescribed by physicians for mentally ill patients (Jackson, 1993). However, it remained highly stereotypical: male patients worked in agriculture and groundskeeping while female patients worked in vegetable and fruit gardens (Moher, 1907). Since wages for patients were not recommended, this work was unpaid, which left the door open to exploitation (Mattei, 2008).

Among the movements that shaped the development of occupational therapy, mental health activism played a critical role in advocating for a reform of mental institutions through the deinstitutionalisation of mentally ill patients and for the regulation of the industrial conditions that produced these illnesses (Frank and Zemke, 2008). Reformist movements in the United Kingdom and United States also included the Arts-and-Crafts, the Moral Treatment and Mental Hygiene movements (Frank, 1992). The goals of these movements were intertwined with socialist and democratic political reforms seeking to prevent not just the alienation and domination of mentally ill patients in asylums, but also the social exclusion and marginalisation of populations created by industrial capitalism (Frank and Zemke, 2008). Outside psychiatric asylums, crafts became used as a dis-alienating and meaningful use of time (Mattei, 2008), which included recreation and any form of enjoyment for the disenfranchised, poor immigrant or minority communities in the United States when capitalist industrialism was at its peak (Dickie, 1996). In this context, artisanal work did not just have an aesthetic or recreational purpose; it was also intended to transform and realign the power relations between workers and owners of the means of production (Dickie and Frank, 1996). In the 20th century, crafts production was eventually used as resistance to political domination and oppression, not only by Jews in Nazi concentration camps but also by Palestinians in the West Bank and Gaza under Israeli occupation (Frank, 1996).

In the Global North, the emergence of occupational therapy as a profession using arts and crafts during the First and Second World Wars may nevertheless be viewed as a product of resistance movements being institutionalised to support the rehabilitation of wounded soldiers (Schemm, 1994). Drawing on its contribution to the war effort, occupational therapy expanded rapidly as a discipline during the early 1920s, with nurses building coalitions with social workers, teachers, artisans and activists to support the war wounded (Bryden and McColl, 2003). In the United States and United Kingdom, the history of occupational therapy has often been told in a narrative according to which physicians advocated for the profession to be included as a component of the treatment and rehabilitation of wounded soldiers (Gutman, 1995). However, relying only on this narrative to explain the history of the profession ignores the resistance predating the war effort (Taff and Balubal, 2021) and the agency of women (Frank, 1992), most of whom were in predominantly female professions, such as nursing, teaching and social work, which gained power and autonomy in the midst of world crises. This Western-centred narrative also tends to overshadow other epistemologies emerging from the Global South (Guajardo et al., 2015).

Arts and crafts constituted the core of occupational therapy for a substantial period of time since its foundation (Friedland, 2003; Levine, 1987) until its *domestication* by biomedicine and the evidence-based movement (Bennett and Bennett, 2000) led to the formation of a highly regulated profession (Freeman et al., 2009). The use of arts and crafts became stigmatised and then was almost completely eliminated from practice (Bissell and Mailloux, 1981); very few occupational therapists use this approach today (Harris, 2008; Perruzza and Kinsella, 2010; Thompson and Blair, 1998). They still tend to be sceptical about using arts and crafts and seem to distance themselves from them (Holder, 2001), despite recent interest in reintegrating arts as a method to improve health (Hansen et al., 2021). The way arts and crafts were slowly rejected from occupational therapy could contain a certain amount of violence, including epistemic violence (Holmes et al., 2012), that was institutionalised in the political process governing its practice (Taff and Balubal, 2021). For these reasons, we cannot afford to use neutral terms when discussing this process. While it might seem unusual for a profession like occupational therapy to use politically charged terms, we think they are necessary.

Drawing on the work of poststructuralist philosophers Deleuze and Guattari, our aim in this article is to critically appraise the biomedicalisation of occupational therapy knowledge and practice, while demonstrating how this process represents a form of ‘imperial’ patronage (Klein, 2012), which must be resisted for the future of this discipline. Such a critical appraisal requires deconstructing some of this profession’s most taken-for-granted assumptions, thereby negating aspects assumed to be ‘good’ and ‘natural’, which might run counter to the professional imperative to be positive (Nicholls, 2013). However, deconstructing the discourses and practices that surround occupational therapy is not destructive; it is in fact productive insofar as the result is ultimately positive (Williams, 2005). To help readers less familiar with a Deleuzo-Guattarian perspective, we introduce two concepts central to their work: *schizoanalysis* and royal/nomad science.

## Theoretical background

Respectively introduced in *Anti-Oedipus* (1972) and *A Thousand Plateaus* (1987), the concepts of *schizoanalysis* and royal/nomad science stem from Deleuze and Guattari’s seminal work on *Capitalism and Schizophrenia*. The thinking of philosopher Gilles Deleuze and psychoanalyst Félix Guattari emerged during the French revolts in May 1968 and is still influential today. Foucault (1977) said that ‘One day, perhaps, the century will be called Deleuzian’ (p. 165). Because the work of Deleuze and Guattari may appear ‘unsettling and perplexing, if not downright maddening’ (Holland, 1999, p. 1) to anyone unfamiliar with their approach, we first discuss the theoretical underpinnings of poststructuralism, then provide definitions of the concepts used in this article.

### Poststructuralism

Poststructuralism was developed in the 1960s and 1970s by French continental philosophers, including Gilles Deleuze and Félix Guattari, Jacques Derrida, Michel Foucault, Jean-François Lyotard and Julia Kristeva (Williams, 2005). While their approaches differ in various ways, the development and expansion of the poststructuralist theoretical perspective were more broadly focussed on and critical of the concepts of truth, discourse, knowledge production and power, as well as normalisation processes. Grounded in the paradigm of critical theory (Guba and Lincoln, 1998), poststructuralism became more and more popular in the field of health sciences, with researchers in nursing (Holmes and Gagnon, 2018), physiotherapy (Eisenberg, 2012; Nicholls, 2017) and occupational therapy (Rudman, 2012; Weinblatt and Avrech Bar, 2001) seeking to understand experiences of health and illness by renouncing the received views of science and proposing alternative paths to the dominant biomedical and postpositivist paradigm. Original work drawing on Foucault and the concept of governmentality was conducted in occupational therapy very recently (Farias and Laliberte Rudman, 2016; Rudman and Aldrich, 2016, 2017), but the concepts developed by Deleuze and Guattari were applied more rarely (Araujo Silva and Lima, 2020; Barlott and Turpin, 2021; Barlott et al., 2017; Lima and Pelbart, 2007; Oliveira and Sant’Anna, 2017). We think a Deleuzo-Guattarian perspective could be useful in understanding and criticising concepts central to occupational therapy, such as the interdependence between mind, body and environment, processes of becoming, collective occupational living, difference, multiplicity and identity (Ikiugu, 2007). It is possible to use concepts coined by Deleuze and Guattari to examine the patronage undertaken by biomedicine in occupational therapy and to reveal the political nature of this process (Holmes et al., 2008). To do this, we will conduct a *schizoanalysis* of the process by which the evidence-based movement later became the preferred path for the future of occupational therapy and show how this was nothing more than a trend meticulously constructed by authority figures (Holmes et al., 2007).

### Schizoanalysis

In their first volume *Anti-Oedipus* (1972), Deleuze and Guattari introduced the concept of schizoanalysis as a tool to critique psychoanalysis. By the 1970 and 1980s, psychoanalysis had achieved a certain respectability in psychiatry and psychology. Following a path similar to the evidence-based movement, it created dominant discourses that were translated into theories and concepts, such as the oedipal complex (Deleuze and Guattari, 1972). The prefix ‘schizo’ comes from schizophrenia, which was deemed unresponsive to psychoanalysis, unlike paranoia, which involves a different type of psychological configuration with respect to energy/desire (libido) investment (Holland, 1999). Paranoia is kept enclosed in a predetermined agenda (*univocal*) whereas schizophrenia involves a process of liberating oneself from any form of ‘codification’ (*polyvocal*). The paranoid configuration clearly aligns with social norms (or ‘discipline’, such as occupational therapy), which are often enforced brutally and violently, but are also more subtly imposed using different techniques (audits, inspections, grading, etc.). In contrast, the schizophrenic configuration is more aligned with a will to change the hegemonic order in a permanent cycle of transformation (Holland, 1999).

Both configurations are present in occupational therapy today. On the one hand, paranoia could be conceived of as the school of thought where all knowledge is fixed in regimes of truth while, on the other, schizophrenia expresses a form of meaning characterised by radical fluidity and unexpectedness (Holmes et al., 2007). The evidence-based movement follows a ‘paranoid’ configuration, inasmuch as its aim is to produce truths drawing on a hierarchy of evidence (with systematic reviews and randomised controlled trials topping the list). This movement over-focuses on internal validity rather than external validity or sociohistorical interactions, whereas theoretical and philosophical papers, considered to be lower down on the evidence list, correspond to a ‘schizo’ configuration (Holmes et al., 2007). The rise of the evidence-based movement has many similarities with the upsurge of psychoanalysis. It is precisely when a dominant discourse becomes a ‘totalising’ perspective that schizoanalysis demonstrates its usefulness (Deleuze and Guattari, 1972).

The first (negative) task of schizoanalysis is to deconstruct such dominant discourses while the second (positive) task is to suggest possibilities for resistance (lines of flight) against paralysing discourses in a field such as occupational therapy (Holmes et al., 2007). While the term ‘schizophrenia’ may often be misread, for Deleuze and Guattari (1972) this concept goes beyond psychopathology. By releasing the ‘will-to-something-else’, schizophrenia is more of a revolutionary *breakthrough* than a psychic *breakdown* (Holland, 1999). In this case, the schizophrenic process corresponds precisely to a type of social (political) functioning representing ‘free-form’ interactions between persons and sociopolitical practices. Therefore, the object of schizophrenia is to achieve radical freedom of thought by escaping from the imposed constraints of dominant ideologies through a flow of creative energy (Patton, 2000). According to Deleuze and Guattari, this revolutionary breakthrough is only partial and involves mostly its *potential* rather than its realisation. A complete form of sociopolitical schizophrenia is an unachievable ideal only because its terms are determined by dominant viewpoints, which provokes further resistance (Fiske, 1989). The interaction between dominant discourses and resistance movements is what interests us here.

### Royal science and nomad (minor) science

In their second volume *A Thousand Plateaus* (1987), Deleuze and Guattari described the concepts of royal science and nomad (minor) science based on the historical study of interactions between nomadic peoples and imperial State apparatuses. These concepts will help us show how resistance by nomad science arises in conflict with the (dominant) royal science, imposed by the sovereign, governors and colonial powers. The objective of the royal (State) science is to ‘striate’ the territory over which it reigns, that is, to territorialise it or control access (Deleuze and Guattari, 1987). Like the gates of a city, royal science filtres who comes in and out and refuses access to those who do not conform to predefined norms (Holmes et al., 2008). Because of its ability to regulate ‘good’ and ‘bad’ ways of producing knowledge, royal science legitimises only predefined, fixed norms. Nomad science, on the other hand, produces external forms of knowledge (also called delinquent knowledge), which disrupt the dominant configuration of royal science (Deleuze and Guattari, 1987). Located on the periphery, nomad science is more interested in looking for singularities and multiplicities than in reinforcing a single truth. In addition, nomads are subjected to a way of producing knowledge that requires their obedience, domestication and subordination (Holmes et al., 2008). Because its creative way of working threatens the hegemonic order, nomad science is often repressed, discredited or viewed as ‘barbaric’ (unsophisticated) by the royal science (Deleuze and Guattari, 1987). This leads nomads to *resist* in order to continue to exist.

Because of the violence with which the royal science represses the nomad science, their relationship is one of conflict and confrontation (Deleuze and Guattari, 1987). In occupational therapy, this confrontation is observed in the development of professional standards, which impose themselves as regimes of truths (Turcotte and Holmes, 2021). Faced with the State apparatus, nomads invented ‘war machines’, whose main objective is paradoxically not that of war (Deleuze and Guattari, 1987). War is in fact a secondary object of the war machines; it is mainly a product of constant struggles with the State apparatus (Patton, 1984). Just as a tool is crafted from its specific use in a specific work context, resisting the royal science requires nomads to craft weapons that are adapted to the threat imposed on them. The weapons of war machines are crafted *from* their relationships with the State apparatus rather than *for* the purposes of war (Patton, 1984).

This process can be understood through the concept of ‘nomadology’, which is a treatise on resistance. For Deleuze and Guattari (1987), resistance is a process of ‘becoming minor’, which consists of participating in a movement that escapes the paralysing thought of dominant perspectives. This process can also be called ‘deterritorialisation’, which is a necessary step in understanding the fixed territory of the royal State apparatus (territorialisation). Just as a code can be understood by decoding it, deterritorialisation means ‘decoding’ a territory, as seen by those who try to escape from it. To be freed from the *apparatuses of capture*, nomads have no choice but to build ‘rhizomatic’ relations – decentralised and horizontal – that disrupt the ‘arborescent’ structure of royal science, whose disciplines emerge vertically from a ‘tree-like’ central trunk (Deleuze and Guattari, 1987). One must therefore become minor, be part of a minority and become nomad. Otherwise, the royal science will do everything to prevent nomads from continuing to exist by *appropriating* their war machines (Deleuze and Guattari, 1987). When appropriated by the State, war machines change their nature and function in order to operate against nomads or other States, to the extent that a State imposes its aim upon them.

This appropriation is not without risks and could unfold in two repulsive scenarios (Deleuze and Guattari, 1987). According to Deleuze and Guattari, the first scenario is a form of *microfascism*, which turns war into an unlimited movement with no other aim than itself (i.e. destroying its enemy), while in the second scenario, which may be called *post-fascism*, total war surpasses itself and transforms into an even more terrifying form of peace, that is, the peace of *Terror* or *Survival*. We are well aware that applying Deleuze and Guattari’s concepts to deconstruct professional practices such as occupational therapy may be controversial and should be done with caution, even metaphorically. As queer French-speaking academics in a North American context and in line with our theoretical perspectives, we are not positioned to express views on epistemic violence such as those experienced by colonised populations in the Global South. Rather, we think that concepts aiming to explain some aspects of these ‘colonial’ or imperialistic processes may potentially be useful in resisting epistemic injustices faced by various practitioners globally, not only in the Global South.

The concepts of royal/nomad science complement that of schizoanalysis in deconstructing the process by which biomedicine appropriated the war machine of nomad occupational therapy and perhaps turned it against itself through the exclusive use of occupation-based theories and models. These concepts will give occupational therapy a refreshing (yet critical) philosophical foundation for revisiting our understanding of this complex discipline.

## Deconstructing the biomedicalisation of occupational therapy

Although it includes many contingent historical, linguistic, social and political discourses, the development of occupational therapy has often been told through its ‘natural’ evolution as a health profession in the evidence-based movement. To understand how occupational therapy might evolve differently in the future requires critically examining its most taken-for-granted assumptions that appear ‘necessary’ and ‘true’ and attempting to deconstruct them.

### Domesticating the lost sheep of nursing

In the United States, the most rapid developments in occupational therapy took place during the First and Second World Wars (Gritzer and Arluke, 1985) when a coalition of nurses, along with social workers, teachers and artisans, were deployed as ‘reconstruction aides’ (Hopkins, 1983). The emergence of occupational therapy as a profession in the health domain coincided with the release of new creative energy through the use of arts and crafts as a therapy for wounded soldiers (Bryden and McColl, 2003). As the focus of wartime economies was on productive work and efficiency (Gritzer and Arluke, 1985), practising arts and crafts could be viewed as a form of resistance for an emerging health profession in the medical sector (Frank and Zemke, 2008), which mainly valued ‘repairing’ injured bodies so that soldiers could resume their productivity (Bryden and McColl, 2003). Because the biomedical hegemony was reluctant to employ recreational uses of occupations, reconstruction aides eventually had to use subversive strategies in order to disrupt the *status quo* that kept them subjugated (Frank and Zemke, 2008).

While not all reconstruction aides had a background in nursing (Frank, 1992), many nurses in the United States decided to turn to occupational therapy under the leadership of Tracy (1910), a nurse who published *Studies in Invalid Occupations, A Manual for Nurses and Other Attendants*. In several institutions in Europe, nurses used occupational therapy before it was even called occupational therapy (Mattei, 2008) and this new role generated excitement and gratification as well as greater autonomy and freedom (Cameron, 1911) and better working conditions (Frank, 1992). Often portrayed as a branch of nursing (Dunlop, 1933) or as nurses’ aides (Gutman, 1995), occupational therapy was an escape route for nurses who felt ‘the need of relaxation from the strain of nursing’ (Cameron, 1911, p. 494). In that sense, we argue that occupational therapy can be viewed as a ‘lost sheep of nursing’. The Christian reference is relevant here to understand the escape of the ‘lost sheep’ who resisted the regime of rules imposed by biomedicine. For nurses, that meant escaping from their traditional role of caring for the sick at the bedside to that of activating wounded soldiers using arts and crafts (McPherson, 2003). In military hospitals, occupations progressively moved from the bedside to activities in the community (Schemm, 1994). In addition to freeing nurses from the primary bond that tied them to biomedicine and nursing, these acts of resistance were a revolutionary breakthrough that set the stage for the development of occupational therapy in the Global North (Dunlop, 1933).

As a dominant power, biomedicine was responsible for ensuring the surveillance, policing and control of subjugated allied health professions, including nursing (Holmes et al., 2008) and physiotherapy (Setchell et al., 2018). Similar to a colonial force, the role of the biomedical power was to territorialise the space over which it reigned. Because reconstruction aides were less subject to biomedical surveillance, their agency increased (Frank, 1992), leading them to undertake a deterritorialisation process. In other words, this allowed many nurses together with social workers and mental health activists to escape from the patronage of biomedicine (Cameron, 1911) and move towards unknown territories or, using Deleuze and Guattari’s terminology, to ‘become minor’. Before it was institutionalised as a health profession, occupational therapy increased its knowledge through resistance to the domination of industrialism and reductionist science (Taff and Balubal, 2021). The aim of using arts and crafts was to reform institutions by producing external forms of knowledge with populations who were excluded and marginalised. Early occupational therapy could thus be conceptualised, in Deleuzo-Guattarian terms, as a ‘nomad science’. Benefitting from the invisibility provided by world crisis contexts in which female-dominated professions could develop outside the control of authority figures (Frank, 1992), these occupational therapists were able to exist peripherally to the biomedical power (Schemm, 1994). As nomads do when confronting a royal science, occupational therapy created its own ‘weapons’ through arts and crafts so that it could exist creatively and aesthetically outside the control of biomedicine (Levine, 1987).

### The imperial patronage of biomedicine

Arts and crafts emerged not only as a critique of medical reductionism (Taff and Balubal, 2021); this movement has also been in constant conflict with it throughout the development of occupational therapy as a profession (Frank, 1992). If early occupational therapy is considered a nomad science, then biomedicine may be conceptualised as a ‘royal science’ trying to control access to the health domain (Deleuze and Guattari, 1987). The conflict between royal and nomad science has been observed through the development of professional standards and norms, the enforcement of accreditation processes and the privatisation of higher education (Turcotte and Holmes, 2021). From the beginning of the First World War, physicians were responsible for dictating the roles and functions that occupational therapy would have in the health sector and were highly invested in the creation of schools where they established qualification standards and curricula centred around medical disabilities, physiology and anatomy (Gutman, 1995). Along the lines of the mind-body dichotomy (Schemm, 1994), the influence of the biomedical model was subsequently observed with reference to the body-as-machine, the need for a basic science in occupational therapy, the conception of occupational therapy as a medical and not a social discipline and the emphasis on work rather than holistic aspects of occupations (Bryden and McColl, 2003). The diversional and recreational use of arts and crafts increasingly competed with their therapeutic and medical functions (Levine, 1987). These purposes frequently overlapped, so much so that it was difficult to separate the therapeutic from the aesthetic ideology of the arts-and-crafts movement (Frank and Zemke, 2008). Debates on the diversionary versus therapeutic purpose of arts-and-crafts activities became more and more prevalent during the 1930s, which slowed the development of the discipline (Bryden and McColl, 2003). During the Second World War, the scientific reductionism of biomedicine forced occupational therapy to further revise its practice (Frank and Zemke, 2008), in particular when the rehabilitation paradigm induced a focus on more functional aspects of occupations (Kielhofner and Burke, 1977).

Despite the freedom that characterised its early years, occupational therapy was soon caught in a new cycle of biomedical patronage (Rogers, 1982). The more visible occupational therapists became, the easier it was for the biomedical model to conduct active surveillance and to control and police them. As Foucault (1995) stated, ‘visibility is a trap’ (p. 200). As it did with nursing (Holmes et al., 2008) and physiotherapy (Nicholls, 2021), the biomedical model embarked on the ‘imperial’ patronage of occupational therapy (Klein, 2012), with the same objective to territorialise it, that is, control who enters and leaves the field of health sciences. Unless they could provide evidence of their effectiveness, occupational therapists would be asked to abandon arts-and-crafts activities as these would not conform to fixed, predefined norms that only the biomedical model could determine (Bissell and Mailloux, 1981), drawing on a dominant post-positivist paradigm (Taff and Balubal, 2021). To obtain recognition and validation as health professionals, occupational therapists were forced to conform to a regime of norms imposed by the evidence-based discourse in the 1990s (Ottenbacher et al., 2002), which was greatly influenced by the biomedical model (Holmes et al., 2006). The aim was for occupational therapists to develop knowledge that would meet the highest standards in the pyramid of evidence, with systematic reviews and randomised controlled trials at the top (Tomlin and Borgetto, 2011). Because of the intrinsically creative and highly complex nature of arts and crafts, these activities could not be assessed under controlled conditions and were reduced to their simplest form, if not disqualified outright (Thompson and Blair, 1998).

Considering the symbolic violence of this patronage, occupational therapists who persisted in using recreational occupations had no choice but to resist in order to survive (Youngson, 2019). Thus, we argue that early occupational therapy activated a form of nomad war machine in response to the repression of the biomedical model (Bryden and McColl, 2003). This exemplifies the logic of *Terror* or *Survival* that nomad war machines face when confronting a royal science that threatens to appropriate them (Deleuze and Guattari, 1987). The recent recognition of and interest in ‘social prescriptions’ could be viewed as a biomedical uptake of the arts-and-crafts movement dear to occupational therapists (Thew et al., 2017). While arts and crafts were originally conceptualised as collective occupations that would liberate and benefit the group as a whole in a non-prescriptive fashion (Levine, 1987), this recent instantiation does not have such aims (Husk et al., 2019). Instead, it is seen as a personalised form of treatment centred on the diversional use of occupations, but only as a short-term biomedical treatment for mental illness, not for the pleasure it may provide (Pescheny et al., 2018). As such, it serves neoliberal interests by reintroducing and imposing an individualistic and paternalistic form of therapy (Gibson et al., 2021), thereby denaturalising the arts-and-crafts movement. In that sense, it is one example of a war machine appropriated by the imperial State apparatus, which could turn against other nomads or any other State. According to Deleuze and Guattari (1987), this appropriation might be more harmful than beneficial.

### Imposing a new regime of truth

As it endeavoured to become an independent profession, occupational therapy was not only territorialised by external biomedical discourses, it was also controlled from within by imposing its own occupation-based models/theories to distinguish it from other professions (Reid et al., 2019). In this, occupational therapy followed a similar path to nursing (Holmes et al., 2008; Traynor, 2009). Drawing on occupational science as a collateral basic science (Clark et al., 1991), the occupational jargon (occupational justice, occupational rights, occupational balance, etc.) imposed itself across academia, university curricula, research and practices (Hooper et al., 2016). This position prevails across most leading organisations within the profession (Vallée, 2020), also endorsing the evidence-based movement as the preferred path for the evolution of its practice (Morley et al., 2011) and the development of the discipline. However, this jargon has imposed its own regime of truths (Hammell, 2009). By making the evidence-based movement the chosen path for the future of the profession, occupational therapy followed a paranoid configuration (Deleuze and Guattari, 1972). Again, this behaviour has a lot in common with the development of nursing (Holmes et al., 2007) and other disciplines enthusiastic about evidence-based discourse (Crosbie, 2013). It is kept enclosed in a predetermined agenda, aligning with disciplinary norms imposed by occupational theories/models.

This trend imposed itself globally in a fairly imperialistic manner (Hammell, 2011), as has been observed by Guajardo Cordoba (2020) and Emery-Whittington (2021) with respect to the controversial use of the concept of ‘occupational justice’. Criticised for being overly individualistic and widely dominated by Western, Anglo-Saxon, female, middle-class and heteronormative perspectives (Trentham et al., 2006), it can be argued that occupation-based theories/models also act as a royal science in relation to other forms of ‘nomad’ occupational therapies developed in the Global South (Dos Santos and Spesny, 2016; Guajardo et al., 2015). The presence of colonial and imperialistic processes in occupational therapy education and theoretical models has subsequently been criticised for reproducing the patronage that rules unequal North-South relations (Grenier, 2020; Hunter and Pride, 2021), leading other scholars to advocate for the decolonisation of the discipline (Huff et al., 2020; Simaan, 2020). Consistent with the principles of evidence-based medicine (Sackett, 1997), the profession had to impose its own set of norms with which occupational therapists around the world are required to conform (Hammell, 2009). While consistently trying to reject biomedicine’s regime of truth (Rogers, 1982; Wilding, 2011), occupational therapy created one of its own by promoting the exclusive use of Western occupation-based knowledge and subsequently imposing it on the Global South and elsewhere (Hammell, 2011). Occupational therapy is now facing another political threat, one which will come from within the profession.

The imperialism underlying the imposition of occupational theories/models is more difficult to challenge since the terms under which any critique could occur are controlled by those in a position of authority (Hammell, 2009). The main reason why this presumptive ‘good’ is so difficult to challenge is that it seems impossible to levy an ‘internal’ critique from within a closed system (Turcotte and Holmes, 2021). Given the apparent consensus related to the occupational jargon (Hammell, 2011), occupational therapists must respond with a vigilant resistance. As this dominant discourse becomes a ‘totalising’ perspective in occupational therapy, schizoanalysis is one of the tools available to support its deconstruction (Deleuze and Guattari, 1972). As a first task of schizoanalysis, we can deconstruct the process by which the occupational jargon was constructed by authority figures based on contingent sociopolitical factors (Hammell, 2011). While this dominant discourse is the response of a profession that wanted to be distinct from other professions, it is also based on efforts to ‘return to the roots’ of occupational therapy by focussing exclusively on occupational concepts (Ikiugu, 2007), illustrating the arborescent structure of the royal science. This process occurred during the 1950s and 1960s and then again in recent years with the creation of doctorate degrees in occupational science, primarily in the United States (Clark et al., 1991), which sanctioned these dominant discourses and dismissed alternative ways of doing, being and thinking about occupations (Turcotte and Holmes, 2021). However, just about anything could have the label ‘occupational’ added and be appropriated by the regime, as if by royal scientific decree. This would correspond to a reterritorialisation by the *apparatus of capture* of occupational science or be a way to reduce and eliminate (nomad) occupational therapies by reproducing the methods of (royal) biomedicine. In order to wage an ‘external’ critique of dominant discourses inside the profession, occupational therapists may need to engage in deterritorialisation strategies and perhaps reactivate ‘war machines’.

## Reactivating the nomad ‘war machines’

Before being appropriated by State apparatuses during the war efforts, the reformist movements that led to occupational therapy invented their own war machines to resist medical reductionism and the industrialisation of society (Frank and Zemke, 2008). If the escape of the lost sheep of nursing could be described as nomadic resistance, what followed looked more like the appropriation of a war machine by the imperial State apparatus. Throughout the development of the profession, the patronage of biomedicine imposed a paranoid configuration using strict norms based on reductionist science and evidence-based medicine; this configuration was pursued in the exclusive use of occupation-based knowledge and its further imposition on the Global South (Dos Santos and Spesny, 2016). As a second task of schizoanalysis (Deleuze and Guattari, 1972), we must seek ways to resist and escape the paranoia that shapes the *status quo* in occupational therapy. Because both the biomedical model and occupational theories/concepts try to impose a rigid agenda for knowledge development in occupational therapy, resistance is required on both fronts (Holmes et al., 2007). This means, first, resisting the biomedical hegemony in the evidence-based movement and, second, avoiding the establishment of a rigid roadmap within occupational therapy through the imposition of occupation-based models/theories in reaction to its patronage by biomedicine (Holmes et al., 2008).

Drawing on the concept of ‘nomadology’ (resistance), we suggest that occupational therapists could use subversive strategies to escape (deterritorialise) the yoke of biomedicine and constitute a war machine. We realise that using politically charged terms for conceptualising this resistance might appear strange for a seemingly harmless profession like occupational therapy. We are using them in this case to illustrate a critical response equivalent to the violence of the patronage and appropriation imposed on them by the royal science of biomedicine (Holmes et al., 2008). War machines are just one way to disrupt the hegemonic order that codifies the evolution of occupational therapy in a predetermined agenda (Deleuze and Guattari, 1987). To reactivate a war machine, occupational therapists can turn to their history and accept how resistance contributed to their becoming (Frank and Zemke, 2008). Even if the beginnings of occupational therapy looked more like a schizophrenic configuration (Deleuze and Guattari, 1972), this configuration was rapidly and insidiously converted into a more paranoid one. To avoid perpetuation of the paranoid configuration, what is needed is a release of new creative energy and free-form interactions similar to those which prevailed when the war machine produced its first revolutionary breakthrough. This requires expressing a radical fluidity, unexpectedness and a ‘will-to-something-else’ (Holland, 1999).

Because such an endeavour would subject them to repression by the royal State apparatus, occupational therapists could resist by forming *rhizomatic* – decentralised and horizontal – relations (Deleuze and Guattari, 1987). They could strive to create connections with other groups suffering from and resisting biomedical patronage, including nurses, other allied health professions, support workers, patient groups and activists facing various forms of exclusion and marginalisation (Guajardo et al., 2015). For a movement to resist the violence of the colonial force, it must be aware of power imbalances within such a movement that could replicate the methods of the royal science. Its aim is to escape from the arborescent and vertical structure of the State apparatus in order to create coalitions where power is decentralised and shared horizontally (Deleuze and Guattari, 1987). In the Global South, some ‘occupational therapies’ have developed *rhizomatically* in response to Western imperialism and the Cold War (Guajardo et al., 2015), including ‘Social Occupational Therapy’ (Malfitano et al., 2014) and ‘Occupational Therapies Without Borders’ (Sakellariou and Pollard, 2016). Learning from the experience of reformist movements from which they emerged, occupational therapists who work to create free-form connections between people and enable group and collective occupational movements should not be surprised if they experience significantly violent repression: this is how royal science has always operated when confronted with nomads. Therefore, occupational therapy should not be distracted from its revolutionary impulse if it is to remain true to its political roots (Frank and Zemke, 2008) and constitute a war machine through spirited resistance. Suffering from a weak professional identity (Turner and Knight, 2015), occupational therapy recently attempted to dissociate itself from any prior colonial background and claim to be original by returning to its ‘roots’ (Ikiugu, 2007). However, we think there is nothing wrong with looking at occupational therapy within the broader history of nursing and other allied health disciplines, without the resistance of which occupational therapy would have never existed. In fact, we believe that this historical perspective could enrich, not impoverish, the discipline of occupational therapy.

## Conclusion

Occupational therapy has experienced multiple transformations throughout its history, from its domestication as the lost sheep of nursing to its imperial patronage by biomedicine and occupation-based theories/models. Because of the violence (including epistemic violence) inherent in such transformations, this history cannot remain unchallenged or reported using neutral terms; it should pave the way for a pointed critique. In this article, we critically examined the biomedicalisation of occupational therapy knowledge and practice and showed how resistance to this process is essential to the future of the profession. We attempted to demonstrate how occupational therapy was not invented from scratch but was crafted through a series of contingent events within resistance movements and the history of other health disciplines, including nursing.

Because nurses were kept in a subjugated position vis-à-vis biomedicine, the use of arts and crafts in the health domain symbolised a form of resistance. Through resistance, the lost sheep of nursing had to become minor, activate a war machine and metamorphose into occupational therapists; this marked the beginning of their history in the Global North. To domesticate the lost sheep, biomedicine required occupational therapy to abandon the ‘caring’ nature of nursing and focus more on the ‘curing’ nature of the biomedical model. The evidence-based discourse that furthered this patronage forced occupational therapy to circumscribe its practice and limit it to the ‘therapeutic’ value of occupations; the diversional use of occupations was rejected since it was more complicated to prove its effectiveness. In the same vein, the profession slowly shifted its focus to productive occupations, such as employment and self-care, rather than recreational and collective activities done for the pleasure they provide. Not only has this evolution been meticulously constructed by symbolic authority figures to make it sound ‘necessary’ or ‘true’ (Hammell, 2009), it also depends on contingent sociopolitical discourses that impose a rigid agenda for knowledge development in occupational therapy. We argue that the occupational jargon continued this imperialism and *appropriation* of the nomad science in the Global South, just more subtly and insidiously. In our view, the future of occupational therapy should not mean trying to behave according to the terms established by biomedicine and evidence-based discourse, using a jargon that imposes its own regime of truth. As a discipline, occupational therapy must either craft its own terms using subversive and delinquent practices or remain the lost sheep of nursing.

In the midst of another global crisis – the ecological crisis – the contemporary focus on productive – but also damageable and destructive – occupation is no longer acceptable (Persson and Erlandsson, 2002). We should again respond with vigilant resistance and perhaps reactivate war machines by releasing new creative energy similar to the use of arts and crafts to challenge the biomedical model. Because of the violence with which arts and crafts were erased from occupational therapy, bringing back more leisure activities and showcasing them in its practice would be a radical act of political resistance (Shaw, 2001). While it might seem unsettling, theorising this resistance using politically charged terms such as ‘nomadology’ and ‘war machines’ should be central to the discussion to enable occupational therapy to move beyond taken-for-granted assumptions and challenge the assumed goodness of the current *status quo*. What needs extensive knowledge development is perhaps not the ability to promote recreational occupations so much as the ability to effectively resist the discourses that prevent (nomad) occupational therapists and other health professionals from practising as professionals in their own right. In that regard, history certainly has something to teach us.
